# Supplementary material for: In Vitro Hepatotoxicity of Routinely Used Opioids and Sedative Drugs
Source: Curr Issues Mol Biol. 2024 Mar 30;46(4):3022–38. doi: 10.3390/cimb46040189 (PMC11049542; doi:10.3390/cimb46040189)
Supplement: Supplementary file 1 [file cimb-46-00189-s001.zip › cimb-2850469-supplementary.pdf]

Test parameter	0.9 % DMSO	Negative Control
Cell Count (x 1.000) n=3	730 (605 - 1020)	555 (435 – 610)
Vitality [%] n=3	80 (80 – 89)	84 (79 – 89)
LDH [U/l] n=5	71 (44 – 81)	25 (21 – 30)
XTT (OD) n=15	1.8 (1.8 – 2.8)	1.3 (1.2 – 1.5)
CYP1A2-activity: resorufin [pmol/l], n=8	3.7 (2.9 – 4.8)	5.5 (2.6 – 5.7)

Supplementary Table S1: Test results for HepG2/C3A cells after exposure to 0.9 % DMSO (dimethyl sulfoxide) compared with pure cell culture medium after 3 days incubation. Values represent as median and min/max. LDH: lactate dehyrogenase

	pH		
	C_{max}	C_{5x}	C_{10x}
**Midazolam (300 ng/ml│1500 ng/ml│3000 ng/ml)**	7,71<br>7,71 / 7,75	7,71<br>7,69 / 7,73	7,82<br>7,78 / 7,82
**Propofol (2 µg/ml│10 µg/ml│20 µg/ml)**	7,69<br>7,69 / 7,71	7,73<br>7,73 / 7,75	7,69<br>7,67 / 7,69
**S-Ketamine (1 µg/ml│5 µg/ml│10 µg/ml)**	7,70<br>7,70 / 7,71	7,79<br>7,78 / 7,80	7,81<br>7,80 / 7,82
**Thiopental (1 mg/ml│5 mg/ml│10 mg/ml)**	7,75<br>7,75 / 7,77	7,79<br>7,79 / 7,83	8,01 *<br>7,92 / 8,01
**Fentanyl (10 ng/ml│50 ng/ml│100 ng/ml)**	7,74<br>7,72 / 7,76	7,68<br>7,66 / 7,70	7,68<br>7,68 / 7,68
**Remifentanil (15 ng/ml│75 ng/ml│150 ng/ml)**	7,66<br>7,65 / 7,66	7,62<br>7,60 / 7,65	7,62<br>7,62 /
**Sufentanil (1 ng/ml│5 ng/ml│10 ng/ml)**	7,66<br>7,65 / 7,68	7,75<br>7,73 / 7,78	7,71<br>7,70 / 7,72
**Negative control**	7,75<br>7,71 / 7,85		

Supplementary Table S2: pH for HepG2/C3A cells in medium after exposure to sedatives and opioids in concentrations C_{max}, C_{5x} and C_{10x}. Values represent as median and 25th/75th percentile. Significance between negative control and exposure groups is indicated by * $p < 0.05$.

	pH		
	C_{max}	C_{5x}	C_{10x}
**Midazolam (300 ng/ml | 1500 ng/ml | 3000 ng/ml)**	7,63<br>7,61 / 7,65	7,88 *<br>7,79 / 7,89	7,74<br>7,71 / 7,74
**Propofol (2 µg/ml | 10 µg/ml | 20 µg/ml)**	7,64<br>7,63 / 7,65	7,68<br>7,67 / 7,69	7,68<br>7,64 / 7,68
**S-Ketamine (1 µg/ml | 5 µg/ml | 10 µg/ml)**	7,64<br>7,64 / 7,65	7,74 *<br>7,72 / 7,74	7,73 *<br>7,71 / 7,74
**Thiopental (1 mg/ml | 5 mg/ml | 10 mg/ml)**	7,71 *<br>7,70 / 7,71	7,95 *<br>7,84 / 7,97	7,76 *<br>7,74 / 7,77
**Fentanyl (10 ng/ml | 50 ng/ml | 100 ng/ml)**	7,67<br>7,66 / 7,67	7,65<br>7,64 / 7,65	7,64<br>7,63 / 7,68
**Remifentanil (15 ng/ml | 75 ng/ml | 150 ng/ml)**	7,58<br>7,58 / 7,59	7,64<br>7,61 / 7,76	7,58<br>7,56 / 7,58
**Sufentanil (1 ng/ml | 5 ng/ml | 10 ng/ml)**	7,63<br>7,60 / 7,64	7,64<br>7,63 / 7,64	7,63<br>7,60 / 7,67
**Negativkontrolle**	7,66<br>7,60 / 7,69		

Supplementary Table S3: pH for HepG2/C3A cells in plasma after exposure to sedatives and opioids in concentrations C_{max}, C_{5x} and C_{10x}. Values represent as median and 25th/75th percentile. Significance between negative control and exposure groups is indicated by * $p < 0.05$
